# A genome-wide detection of copy number variations using SNP genotyping arrays in swine

**DOI:** 10.1186/1471-2164-13-273

**Published:** 2012-06-22

**Authors:** Jiying Wang, Jicai Jiang, Weixuan Fu, Li Jiang, Xiangdong Ding, Jian-Feng Liu, Qin Zhang

**Affiliations:** 1 Key Laboratory of Animal Genetics, Breeding and Reproduction, Ministry of Agriculture, National Engineering Laboratory for Animal Breeding, College of Animal Science and Technology, China Agricultural University, Beijing, 100193, China; 2 Shandong Provincial Key Laboratory of Animal Disease Control and Breeding, Institute of Animal Science and Veterinary Medicine, Shandong Academy of Agricultural Sciences, Jinan, 250100, China

**Keywords:** Copy number variations, Genetic variation, SNP arrays, Quantitative real time PCR, Pig

## Abstract

**Background:**

Copy Number Variations (CNVs) have been shown important in both normal phenotypic variability and disease susceptibility, and are increasingly accepted as another important source of genetic variation complementary to single nucleotide polymorphism (SNP). Comprehensive identification and cataloging of pig CNVs would be of benefit to the functional analyses of genome variation.

**Results:**

In this study, we performed a genome-wide CNV detection based on the Porcine SNP60 genotyping data of 474 pigs from three pure breed populations (Yorkshire, Landrace and Songliao Black) and one Duroc × Erhualian crossbred population. A total of 382 CNV regions (CNVRs) across genome were identified, which cover 95.76Mb of the pig genome and correspond to 4.23% of the autosomal genome sequence. The length of these CNVRs ranged from 5.03 to 2,702.7kb with an average of 250.7kb, and the frequencies of them varied from 0.42 to 20.87%. These CNVRs contains 1468 annotated genes, which possess a great variety of molecular functions, making them a promising resource for exploring the genetic basis of phenotypic variation within and among breeds. To confirmation of these findings, 18 CNVRs representing different predicted status and frequencies were chosen for validation via quantitative real time PCR (qPCR). Accordingly, 12 (66.67%) of them was successfully confirmed.

**Conclusions:**

Our results demonstrated that currently available Porcine SNP60 BeadChip can be used to capture CNVs efficiently. Our study firstly provides a comprehensive map of copy number variation in the pig genome, which would be of help for understanding the pig genome and provide preliminary foundation for investigating the association between various phenotypes and CNVs.

## Background

Copy number variation (CNV) is defined as a segment of DNA that is 1kb or larger and present at a variable copy number in comparison with a reference genome [1,2]. So far, CNV has gained considerable interests as a source of genetic variation in many species. Extensive studies have been performed to identify and map CNV in humans [1-3], model organisms [4-6] and domestic animals [7-11]. Compared with the most frequent SNP marker, CNVs cover wider genomic regions in terms of total bases involved and have potentially larger effects by changing gene structure and dosage, alternating gene regulation, exposing recessive alleles and other mechanisms [12,13]. CNVs have been shown to be important in both normal phenotypic variability and disease susceptibility [1,13,14] and association studies of CNVs and diseases have become popular in human [15-17]. Additionally, in animals, phenotype variations caused by CNVs were also observed, for instance, the white coat phenotype in pigs caused by the copy number variation of the *KIT* gene [18,19] and the pea-comb phenotype in chickens caused by the copy number variation in intron 1 of the *SOX5* gene [20]. These demonstrate that CNVs can be considered as promising markers for some economically important traits or diseases in domestic animals. Thus, comprehensive identification and cataloging of CNVs will greatly benefit functional analyses of genome variation.

Although pig is one of the most economically important worldwide livestock as well as a suitable animal model for human disease, few studies are focused on investigating CNV in pig compared to other species [4-8,21,22]. So far, there are merely two studies on pig CNV detection reported. Fadista et al. [9] addressed the first account of CNV survey (37 CNVRs) among 12 Duroc boars using a custom tiling oligonucleotide array CGH approach. Ramayo-Caldas et al. [10] identified 49 CNVRs in 55 animals from an Iberian x Landrace cross using Porcine SNP60 BeadChips. Previous studies at genome scale suggest that CNVs comprise up to ~12%, 4% and 4.6% of human[2], dog[21] and cattle [8] genome sequence, respectively. Compared with abundance of CNVRs detected in other species, CNVs detected in pig is far from saturation.

Currently, CNVs can be identified using different technological approaches. Two major platforms, i.e., comparative genomic hybridization (CGH) array and SNP genotyping array, were extensively compared by Redon et al. [2]. Although CGH array based approach has excellent performance in signal-to-noise ratios, the SNP genotyping array has the advantage of performing both genome-wide association studies (GWAS) and CNV detection [23]. CGH arrays report only relative signal intensities, whereas SNP arrays collect normalized total signal intensity (Log R ratio - LRR) and allelic intensity ratios (B allele frequency - BAF) which represent overall copy numbers and allelic contrasts [23]. SNP arrays use less sample per experiment compared to CGH arrays, and it is a cost effective technique which allows users to increase the number of samples tested on a limited budget [24]. Nowadays, SNP arrays have been routinely used for CNV detection in human and other organisms [2,8,10,25], and manufacturers of SNP genotyping arrays have incorporated non-polymorphic markers into their SNP genotyping arrays to improve the coverage of SNP arrays for CNV analyses [26].

In the present study, using the PennCNV software [27], a genome-wide CNV detection based on the Porcine SNP60 BeadChip was performed in a large sample of 474 pigs from four breed populations with different genetic background. Our study firstly provides a comprehensive map of CNVs in the pig genome, which would be helpful for understanding the genomic variation in the pig genome and provide preliminary foundation for investigating the association between various economically important phenotypes and CNVs.

## Results

### Genome-wide detection of CNVs

**Figure 1:**
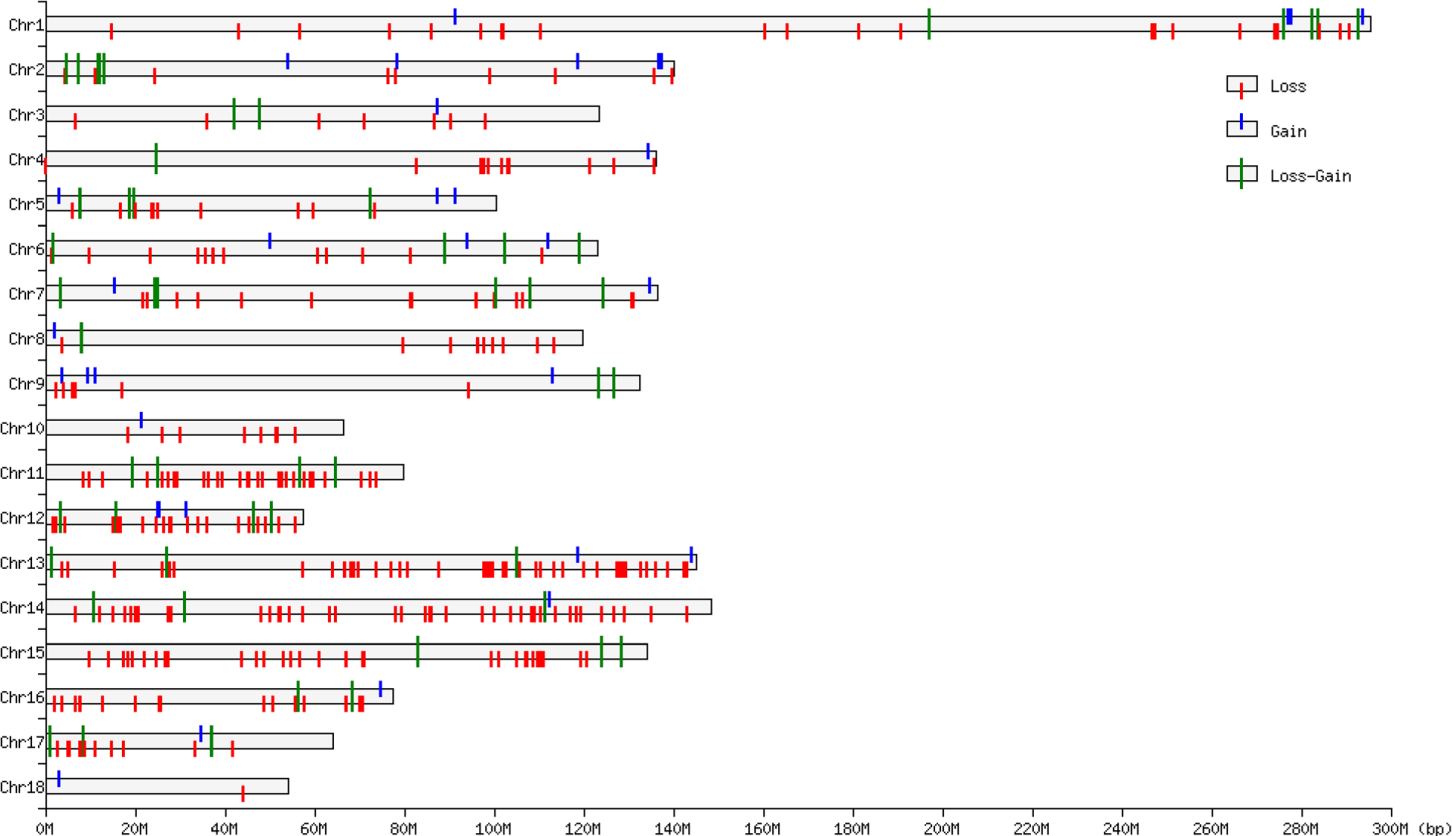
**Genomic distribution of CNVRs in 18 pairs of autosomal chromosomes of pigs.** The chromosomal locations of 382 CNVRs are indicated by lines. Y-axis values are chromosome names, and X-axis values are chromosome position in Mb, which are proportional to real size of swine genome sequence assembly (9.0) (http://www.ensembl.org/Sus_scrofa/Info/Index).

Overall, 4,279 CNVs were assessed by PennCNV on 18 pairs of autosomal chromosomes. The average number of CNVs per individual was 9.03. By aggregating overlapping CNVs, a total of 382 CNVRs (Additional file 1; Table S1) across genome were identified, which cover 95.76Mb of the pig genome and correspond to 4.23% of the autosomal genome sequence. Among these CNVRs, we found 296 loss, 34 gain and 52 both (loss and gain within the same region) events. The length of these CNVRs ranged from 5.03 to 2,702.7kb with a mean of 250.7kb and a median of 142.9kb. The frequencies of these CNVRs ranged from 0.42 to 20.87%. In particular, there were 46 CNVRs with frequency >5%, and 8 CNVRs >10%. Figure 1 summarizes the location and characteristics of all CNVRs on autosomal chromosomes. It is obvious that these CNVRs are not uniformly distributed among different chromosomes. The proportion of CNVRs on the 18 pairs of autosomal chromosomes varies from 2.36-12.04%. Chromosome 13 harbors the greatest number (46) of CNVRs, whereas chromosome 12 has the densest CNVRs with an average distance of 1,226.94kb between CNVRs.

In this study, samples of four populations, including 119 Yorkshire pigs, 13 Landrace pigs, 15 Songliao Black pigs and 327 the Duroc × Erhualian crossbred pigs, were used. Large difference of CNVR numbers were found among the four populations (Table 1). In the Duroc × Erhualian crossbred, we identified 239 CNVRs, which comprised 62.57% of the total CNVRs detected herein. In Yorkshire, 178 CNVRs were detected, corresponding to nearly half of the total number (46.60%), while only 89 (23.30%) and 101 (26.44%) CNVRs were found in Landrace and Songliao Black, respectively. 248 unique CNVRs, i.e., CNVRs detected only in one population, were detected, including 184, 57, 3 and 4 in Duroc × Erhualian crossbred, Yorkshire, Landrace and Songliao Black, respectively.

**Table 1:** Sample sizes and the CNVR numbers detected in the four populations

**Breed**	**Sample size**	**CNVRs number^a^**	**Unique CNVRs^b^**
Yorkshire	119	178	57
Landrace	13	89	3
Songliao Black	15	101	4
Duroc × Erhualian crossbred	327	239	184
Total	474	382	248

^a^ CNVRs number means the total number found in one breed.

^b^ Unique CNVR means CNVR only detected in one breed.

### Gene content of pig CNVRs

Totally, 1,468 genes within the identified CNVRs were retrieved from the Ensembl Genes 64 Database using the BioMart data management system [28], including 1,322 protein-coding genes, 80 miRNA, 29 pseudogenes, 29 snoRNA, 40 snRNA, 11 rRNA, six miscRNA and one retrotransposed gene (Additional file 1; Table S2). These genes are distributed in 282 (73.8%) CNVRs, while the other 100 CNVRs do not contain any annotated genes.

In order to provide insight into the functional enrichment of the CNVs, Gene Ontology (GO) [29] and Kyoto Encyclopedia of Genes and Genomes (KEGG) [30] pathway analyses were performed with the DAVID bioinformatics resources [31]. The GO analyses revealed 119 GO terms (Additional file 1: Table S3), of which 23 were statistically significant after Benjamini correction. And the significant GO terms were mainly involved in sensory perception of smell or chemical stimulus, olfactory receptor activity, G-protein coupled receptor protein signaling pathway, cell surface receptor linked signal transduction, and other basic metabolic processes. There were also some enriched charts with marginal significance, which were involved in antigen processing and presentation, MHC class II protein complex, innate immune response and adaptive immune response. The KEGG pathway analyses indicated that the genes in the CNVRs were enriched in eight pathways (Additional file 1: Table S4), of which six were statistically significant after Benjamini correction, i.e., olfactory transduction, systemic lupus erythematosus, linoleic acid metabolism, drug metabolism, arachidonic acid metabolism, and metabolism of xenobiotics by cytochrome P450.

Additionally, 360 QTLs (Additional file 1: Table S5), affecting a wide range of traits, such as growth, meat quality, reproduction, immune capacity and disease resistance, were found in 16 CNVRs by comparing the overlapping of CNVRs with QTLs in the pig QTLdb (Jan 2, 2011, (http://www.animalgenome.org/cgi-bin/QTLdb/SS/index)).

### CNV validation by qPCR

Quantitative real time PCR (qPCR) was used to validate 18 CNVRs chosen from the 382 CNVRs detected in the study. These 18 CNVRs represent different predicted status of copy numbers (i.e., loss, gain and both) and different CNVR frequencies (varied from 0.84 to 18.57%). A total of 37 qPCR assays (Additional file 1: Table S6), i.e. two or three for every CNVR, were performed. Out of the 37 qPCR assays, 21 (56.76%) were in agreement with prediction by PennCNV. When counting the CNVRs, 12 (66.6%) out of the 18 CNVRs (Table 2) had positive qPCR confirmations by at least one PCR assay. The average frequency and size of the 12 confirmed CNVRs were 4.6% and 295.5kb respectively, which were smaller than those of the six unconfirmed ones (8.2% and 1,034.8kb, respectively) (Additional file 1: Table S6).

**Table 2:** Results of quantitative real-time PCR analysis of the 12 confirmed CNVRs

**CNVR_No.**	**Chr.**	**Start**^**a**^	**End**^**a**^	**Primer_ID**	**Positive samples**				**Negative samples**				**validated**	**Genes**
					**Frequency**	**Type**	**Number of samples**	**Confirmed samples**	**Confirmed rate**	**Number of sample**	**Confirmed samples**	**Confirmed rate**		
6	1	91513835	91584145	17-2	0.0148	gain	7	7	1.0000	5	0	0.0000	Yes	--
				17-8			7	0	--	5	0	--	No	--
11	1	160653704	160713406	4-1	0.0675	loss	32	32	1.0000	15	1	0.0667	Yes	--
				4-4			32	32	1.0000	15	1	0.0667	Yes	--
20	1	274307988	274570232	5-3	0.0274	loss	13	0	--	11	0	--	No	*C5*
				5-7			13	12	0.9200	11	2	0.18	Yes	*TRAF1*
22	1	276291234	276800847	3-1	0.0738	both	35	31	0.8857	11	1	0.0909	Yes	*ORs*
				3-5			35	35	1.0000	15	6	0.4000	Yes	
161	14	52620825	52720705	6-3	0.0211	loss	10	10	1.0000	11	8	0.7273	Yes	--
				6-6			10	10	1.0000	11	8	0.7273	Yes	--
259	2	4834400	5424602	7-3	0.0759	both	18	16	0.8889	12	6	0.5000	Yes	*EFEMP2*
				7-5			36	31	0.8611	12	6	0.5000	Yes	*DPF2*
276	2	137644872	137915976	15-4	0.0928	gain	8	0	--	4	0	--	No	*ORs*
				15-5			38	26	0.6842	19	9	0.4737	Yes	
				15-6			8	7	0.8750	4	1	0.2500	yes	
285	3	87641425	87711895	16-2	0.0422	gain	20	18	0.9000	12	8	0.6666	Yes	--
				16-5			20	19	0.9500	18	10	0.5556	Yes	--
314	5	59744851	60461526	13-1	0.0084	loss	4	3	0.7500	12	0	0.0000	Yes	*CD4*
				13-5			4	3	0.7500	8	0	0.0000	Yes	*GAPDH*	
325	6	37641317	38387857	8-2	0.0211	loss	10	10	1.0000	8	2	0.2500	Yes	*FTL*	
				8-5			10	10	1.0000	8	2	0.2500	Yes	*IRF3*	
344	7	29445530	29634684	9-4	0.0105	loss	5	0	--	7	0	--	No	-	
				9-6			5	5	1.0000	16	10	0.6250	Yes	-	
373	9	4203824	4364337	2-2	0.0928	loss	28	28	1.0000	17	4	0.2353	Yes	*ORs*	
				2-7			29	29	1.0000	17	2	0.1176	Yes		
Mean									0.9269			0.3182			

^a^ The Sus scrofa assembly (9.0) (http://www.ensembl.org/Sus_scrofa/Info/Index) was used to indicate the position of the CNVRs.

**Figure 2:**
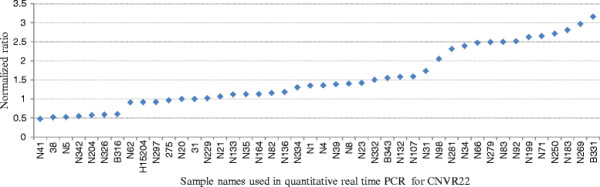
**Normalized ratios (NR) obtained by Quantitative real time PCR (qPCR) for CNVR22.** Y-axis shows the NR values obtained by qPCR, and X-axis represents the sample name of the detected 33 positive and 11 negative control samples. Samples with NR about 1 denote normal individuals (two copy), samples with NR about 0.5 denote one copy loss individuals (one copy), and samples with NR about 1.5 or more denote copy number gain individuals (≧three copy).

**Figure 3:**
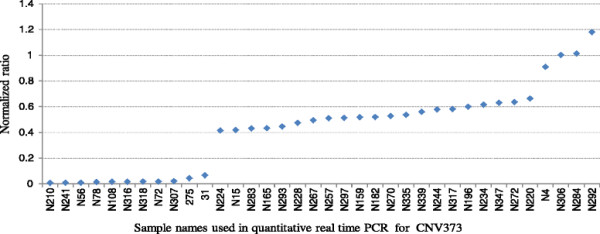
**Normalized ratios (NR) obtained by quantitative PCR (qPCR) for CNVR372.** Y-axis shows the NR values obtained by qPCR, and X-axis represents the sample name of the detected 28 positive and 8 negative control samples. Samples with NR about 1 denote normal individuals (2 copy), and samples with NR about 0 and 0.5 denote one copy and two copies loss individuals (zero and one copy).

For the CNVRs with low frequencies we tested all the positive samples, while for the CNVRs with high frequencies we tested part of them. Furthermore, a certain number of random negative samples were tested as negative control for every CNVR. For the positive samples of the 12 confirmed CNVRs, the proportions of confirmed samples varied from 68.42% to 100%, with an average of 92.69%. For the negative samples of the 12 confirmed CNVRs, the proportions of confirmed samples (i.e. false negative) varied from 0 to 72.73%, with an average of 31.82% (Table 2). Additionally, the copy numbers in some CNVRs varied among individuals. For example, we found one copy loss and different copy gain (three to six copies) in CNVR22 (Figure 2), and one and two copies loss in CNVR373 (Figure 3).

## Discussion

In our study, among the four populations, the largest number of total CNVRs and unique CNVRs were detected in the Duroc × Erhualian crossbred population. In addition to the larger sample size, another important reason is that this population has special genetic background. Particularly, Erhualian is one famous Chinese indigenous breed. Many previous studies have indicated that Chinese indigenous pig breeds have different genetic background with western commercial breeds, such as Duroc, Landrace and Yorkshire [32-35]. Therefore, there are breed-specific CNVs in pigs, which is consistent with the report in cattle [7]. The differences of CNV among breeds supported that some CNVs are likely to generate independently in breeds and therefore, likely contribute to breed differences.

We compared our results with two previous reports on pig CNVs (Additional file 1: Table S7). Ramayo-Caldas et al. [10] firstly used the Porcine SNP60 BeadChip data of 55 animals from an Iberian x Landrace cross to identify CNVs in pig, and detected 49 CNVRs by at least two programs of cnvPartition (Illumina Inc.), PennCNV [27] and GADA [36]. Twenty-two out of the 49 CNVRs (44.9%) are identical or overlapped with our results. Using the custom tiling oligonucleotide array CGH approach, Fadista et al. [9] addressed 37 CNVRs on the SSC4, 7, 14, and 17 of the preliminary assembly of pig genome among 12 Duroc boars. However, only one CNVR of them was found overlapping with our results.

The potential reasons for the different results between this study and the other two studies lie in the following aspects. Firstly, the study population differed in terms of size and genetic background in different studies. A much larger sample size with broader genetic background (three pure breeds and one crossbred population) were included in this study in comparison with the other two studies, where only one breed or crossbreed (different from ours) with very small sample size were involved. Secondly, different platforms, SNP genotyping array and CGH array, are different in the calling technique, resolution difference and genome coverage which contribute to the discrepancy of CNVs detected. Thirdly, previous studies showed that genomic waves have a significant interfere with accurate CNV detection [8,37]. Genomic wave refers to the patterns of signal intensities across all chromosomes, where different samples may show highly variable magnitude of waviness. In our study, the genomic waves were adjusted using the *-gcmodel* option, while it was not in the study of Ramayo-Caldas et al. [10]. The issue of low overlapping rates between different reports was also encountered in CNV studies in other mammal [7,8,38,39].

A large amount annotated genes (1,468 Ensembl genes) are located in the 382 identified CNVRs. The average number of genes per Mb of the 382 CNVRs is 15.32, which is larger than that on the whole genome (9.05) according to the Sscrofa 9.0 assembly in Ensembl (http://asia.ensembl.org/). It has been suggested that CNVs are located preferably in gene-poor regions [40,41], probably because CNVs present in gene-rich regions may be deleterious and therefore removed by purifying selection [42]. In contrast to it, the larger number of genes in the identified CNVRs probably reflects the fact that the Porcine SNP60 BeadChip used in this study is biased toward the gene-rich regions. Functional analyses, such as GO, pathway and overlapping with QTLs in pig QTLdb, suggest that these genes entail a great variety of molecular functions, making them a promising resource for exploring the genetic basis of phenotypic variation within and among breeds. Especially, consistent with CNV studies in human, mouse, cattle, and dog [1,5,7,21], some of the enriched GO terms, such as drug detoxification, innate and adaptive immunity, and receptor and signal recognition, are also present in pigs. Conservation of some CNVs across different species suggests that selective pressure may tend to favor specific gene dosage changes, and genes involved in these CNVs may affect the adaptability and fitness of an organism in response to external pressures [1].

Most of our CNVRs were reported for the first time. In order to confirm these novel CNVRs, we selected 18 CNVRs for validation by qPCR, and 12 of them (66.6%) were validated. The confirmed rate is higher than most of previously reported, such as Fadista et al. [9] in pigs (50%) and Hou et al. [8] in cattle (60%) but a little lower than that reported by Ramayo-Caldas et al. [10] in pigs (71%). In the study of Ramayo-Caldas et al. [10], the CNVRs selected to be validated were detected by at least two programs and were of high frequency, whereas CNVRs selected to be validated herein were detected by one program, with low to high frequencies. The average proportion of the confirmed positive samples of the 12 validated CNVRs were 92.69%, demonstrating that for most of the positive samples qPCR experiments agreed well with the PennCNV prediction, whereas the false negative rate in the negative samples were rather high, with an average of 31.82%. False-negative identification is common in CNV detection, and has been reported previously [9,10,21]. It can be explained by the stringent criteria of CNV detection, i.e., containing three or more consecutive SNPs and presented in at least two individuals, which were applied in order to minimize the false-positive, and thus resulted in high false-negative rate inevitably.

Eight out of the 12 successfully validated CNVRs contain functionally important genes. Three of them (CNVR_ID: 22, 276 and 373) include genes of olfactory receptors (ORs) family. ORs are involved in odorant recognition and form the largest mammalian protein superfamily [43]. Many studies in human and other mammals also indicate that the OR genomic loci are frequently affected by CNVs [2,4,5,40,43,44]. The qPCR assays revealed that all of the three CNVRs could be confirmed by two pairs of primers. The other five CNVRs (CNVR_No 20, 259, 314, 325, 344) contain many important immune-related and basic metabolic genes, including TNF receptor-associated factor 1 (*TRAF1*), EGF containing fibulin-like extracellular matrix protein 2 (*EFEMP2*), D4, zinc and double PHD fingers family 2 (*DPF2*), CD4 molecule (*CD4*), glyceraldehyde-3-phosphate dehydrogenase (*GAPDH*), ferritin, light polypeptide (*FTL*) and interferon regulatory factor 3 (*IRF3*). The functions of these genes have been reported in pig and other species, and their detailed information was showed in Table S9 of the Additional file 1. In particular, *CD4* was the first time to be found to have copy number change not only in pigs but in human and other animals. Considering the important function of genes in them, the five CNVRs are worth to be further studied.

The Porcine SNP60 BeadChip was originally developed for high-throughput SNP genotyping for genome-wide association studies. Although CNV detection is also feasible with such panel, it is impaired by low marker density, non-uniform distribution of SNPs along pig chromosomes and lack of non-polymorphic probes specifically designed for CNV identification [45]. Hence, only large CNVRs are expected to be assessed with the Porcine SNP60 array. Furthermore, the Sscrofa 9 assembly, with 4× sequence depth across the genome, is still in incomplete status, which makes it difficult to determine the boundaries of CNVRs. Accordingly, multiple, neighboring, and discrete CNV events could trigger a larger call by PennCNV, leading to an over-estimation of the CNV size. Therefore, it is quite possible that the qPCR primers used to validate the CNVRs were designed beyond the boundaries of the CNVRs. Besides these aspects, factors, such as potential SNPs and small indels undetected so far, could also influence the hybridization of the qPCR primers in some animals, resulting in unstable quantification values or reducing primer efficiency.

Many gene families, including olfactory receptor, solute carrier, cytochrome P450, MHC and interleukin, which had been reported to be influenced by CNVs in human and other mammals [10,44,46], were also found to be in the CNVRs of this study. Additionally, by converting the pig Ensembl gene IDs to their orthologous human gene, we checked whether they have been included in the Human Database of Genomic Variants (http://projects.tcag.ca/variation/). It turned out that 590 genes (Additional file 1: Table S2), a remarkably high proportion (40.19%) of all the total number genes in the identified CNVRs, were reported to be influenced by CNVs in human.

## Conclusions

We have performed a genome-wide CNV detection based on the Porcine SNP60 genotyping data of 474 pigs and provided the highest resolution CNV map in the pig genome so far. A total of 382 CNVRs were identified. Validating of 18 CNVRs of these CNVRs by qPCR assays produced a high rate (66.67%) of confirmation. We conclude that the currently available genome-wide SNP assays can capture CNVs efficiently. However, it should be noticed that only large CNVRs are expected to be identified using this SNP panel and the number of CNVs identified in this study is likely to be a gross underestimation of the true number of CNVs in the pig genome. Follow-up studies, using improved SNP arrays as well as other technologies, such as CGH arrays and next-generation sequencing [47], should be carried out to attain high-resolution CNV map. Association studies between CNVs and diseases have become popular in human [15-17], and have begun in animal as well [48]. Findings in our study would provide meaningful genomic variation information for association studies between CNV and economically important phenotypes of pigs in the future.

## Methods

### Animal resource

The animals initially used in this study were composed of 1,017 pigs from four populations with different genetic background, including 500 Yorkshire pigs, 85 Landrace pigs, 96 Songliao Black pigs, and 336 Duroc × Erhualian crossbred pigs. Songliao Black is a breed derived from cross of Landrace, Duroc and Min pigs. The Duroc × Erhualian crossbred was formed by crossing eight Duroc boars with 18 Erhualian sows. Both Min pigs and Erhualian pigs are Chinese indigenous breeds.

### SNP array genotyping and quality control

Genomic DNA samples were extracted from ear tissue of all pigs using a standard phenol/chloroform method. All DNA samples were analyzed by spectrophotometry and agarose gel electrophoresis. The genotyping platform used was Infinium II Multisample assay (Illumina Inc.). SNP arrays were scanned using iScan (Illumina Inc.) and analyzed using BeadStudio (Version 3.2.2, Illumina, Inc.). The whole procedure for collection of the ear tissue samples was carried out in strict accordance with the protocol approved by the Animal Welfare Committee of China Agricultural University (Permit number: DK996).

In order to exclude poor-quality DNA samples and decrease potential false-positive CNVs, quality control was performed according to the following procedures. The genome-wide intensity signal must have as little noise as possible. Only those samples with standard deviation of normalized intensity (Log R ratio, LRR) <0.30 and B allele frequency (BAF) drift <0.01 were included. Since wave artifacts roughly correlating with GC content resulting from hybridization bias of low full-length DNA quantity could interfere with accurate inference of CNVs [37], only samples in which the GC wave factor of LRR less than 0.05 were accepted. Finally, 474 samples (119 Yorkshire pigs, 13 Landrace pigs, 15 Songliao Black pigs and 327 Duroc × Erhualian crossbred pigs) with high-quality genotyping (average call rate 99.67%) out of 1,017 samples were remained for CNV detection after quality control.

### Identification of pig CNVs

The PennCNV software [27] was applied to identify pig CNVs in this study. This algorithm incorporates multiple sources of information, including total signal intensity (LRR) and allelic intensity ratio (BAF) at each SNP marker, the distance between neighboring SNPs, the population frequency of B allele (PFB) of SNPs, and the pedigree information where available [27]. Both LRR and BAF were exported from BeadStudio (Illumina Inc.) given the default clustering file for each SNP. The PFB file was calculated based on the BAF of each marker. The SNPs physical positions on chromosomes were derived from the swine genome sequence assembly (9.0) (http://www.ensembl.org/Sus_scrofa/Info/Index). Furthermore, PennCNV also integrates a computational approach by fitting regression models with GC content to overcome “genomic waves”. The pig gcmodel file was generated by calculating the GC content of the 1Mb genomic region surrounding each marker (500kb each side) and the genomic waves were adjusted using the *-gcmodel* option. Although many of the samples had pedigree information initially, most of trio information was unavailable after quality control. So, pedigree/trio information was not incorporated into the analyses.

In this study, CNV was inferred with two criteria: first, it must contain three or more consecutive SNPs, and second it must be present in at least two individuals. Finally, CNVs regions (CNVRs) were determined by aggregating overlapping CNVs identified across all samples according to the criteria proposed by Redon et al. [2].

Due to density limitation of SNPs on chromosome X, i.e. about 86kb of averaged SNP interval, which is two folds of the average interval across whole genome, CNVs detected on chromosome X might have high false-positive rate and were excluded from further analyses in our study.

### Gene contents and functional annotation

Gene contents in the identified CNVRs were retrieved from the Ensembl Genes 64 Database using the BioMart (http://www.biomart.org/) data management system [28]. To provide insight into the functional enrichment of the CNVs, functional annotation was performed with the DAVID bioinformatics resources 6.7 (http://david.abcc.ncifcrf.gov/summary.jsp) [31] for Gene Ontology (GO) terms [29] and Kyoto Encyclopedia of Genes and Genomes (KEGG) [30] pathway analyses. Since only a limited number of genes in the pig genome have been annotated, we firstly converted the pig Ensembl gene IDs to orthologous mouse Ensembl gene IDs by BioMart (Additional file 1: Table S8), then carried out the GO and pathway analyses. Statistical significance was assessed by using *P* value of a modified Fisher's exact test and Benjamini correction for multiple testing.

### Quantitative real time PCR

Quantitative real time PCR (qPCR) was used to validate 18 CNVRs chosen from the 382 CNVRs detected in the study. We used the 2^-ΔΔCt^ method for relative quantification of CNVs [49], which compares the ΔC_t_ (cycle threshold (C_t_) of the target region minus C_t_ of the control region) value of samples with CNV to the ΔC_t_ of a calibrator without CNV. The glucagon gene (*GCG*) is highly conserved between species and has been approved to have a single copy in animals [10,50]. So, one segment of it was chosen as control region. Primers (Table S6 of Additional file 1) were designed with the Primer3 web tool (http://frodo.wi.mit.edu/primer3/). Moreover, the UCSC In-Silico PCR tool (http://genome.ucsc.edu/cgi-bin/hgPcr?command=start) was used for in silico specificity analysis [51]. Prior to performing the copy number assay, we generated standard curves for the primers of target and control regions to determine their PCR efficiencies. To ensure the same amplification efficiencies between target and control primers, the PCR efficiencies for all primers used in the study were required to be 1.95-2.10.

All qPCR were carried out using LightCycler® 480 SYBR Green I Master on Roche LightCycler® 480 instrument following the manufacturer’s guidelines and cycling conditions. The reactions were carried out in a 96-well plate in 20μl volume, containing 10μl Blue-SYBR-Green mix, 1μl forward and reverse primers (10pM/μl) and 1μl 20ng/μl genomic DNA. Each sample was analyzed in duplicates. The second derivative maximum algorithm included within the instrument software was used to determine cycle threshold (C_t_) values for each region.

## Competing interests

The authors declare that they have no competing interests.

## Authors' contributions

WJ carried out gene annotation, experimental validations and wrote the manuscript. JJ carried out computational analysis. LJ and ZQ conceived of the study and led in its design and coordination. JL, FW and DX contributed to the sample genotyping, data analysis and interpretation of data. All authors read and approved the final manuscript.

## Supplementary Material

Additional file 1 **Table S1.** Information of 382 identified CNVRs and their distributions in the four populations. Additional file 1: Table S2. Information of genes in the identified CNVRs and their comparison with Human Database of Genomic Variants. Additional file 1: Table S3. Gene ontology (GO) analyses of genes in the identified CNVRs. Additional file 1: Table S4. Pathway analyses of genes in the identified CNVRs. Additional file 1: Table S5. Previously reported QTLs overlapped with identified CNVRs. Additional file 1: Table S6. Information and the primers used in qPCR analyses of the 18 CNVRs chosen to be validated. Additional file 1: Table S7. Comparison between identified CNVRs and those of previous reports of pig CNVs. Additional file 1: Table S8. Pig Ensembl gene IDs and their orthologous mouse IDs. Additional file 1: Table S9. Functions of the genes validated to be copy number variable by qPCR assay [52-65]. Click here for a file

## References

[B1] Feuk L Carson AR Scherer SW Structural variation in the human genome Nat Rev Genet 2006 7 2 85 97 16418744 10.1038/nrg1767

[B2] Redon R Ishikawa S Fitch KR Feuk L Perry GH Andrews TD Fiegler H Shapero MH Carson AR Chen W Cho EK Dallaire S Freeman JL González JR Gratacòs M Huang J Kalaitzopoulos D Komura D MacDonald JR Marshall CR Mei R Montgomery L Nishimura K Okamura K Shen F Somerville MJ Tchinda J Valsesia A Woodwark C Yang F Zhang J Zerjal T Zhang J Armengol L Conrad DF Estivill X Tyler-Smith C Carter NP Aburatani H Lee C Jones KW Scherer SW Hurles ME Global variation in copy number in the human genome Nature 2006 444 7118 444 454 17122850 10.1038/nature05329 PMC2669898

[B3] Komura D Shen F Ishikawa S Fitch KR Chen W Zhang J Liu G Ihara S Nakamura H Hurles ME Lee C Scherer SW Jones KW Shapero MH Huang J Aburatani H Genome-wide detection of human copy number variations using high-density DNA oligonucleotide arrays Genome Res 2006 16 12 1575 1584 17122084 10.1101/gr.5629106 PMC1665641

[B4] Cutler G Marshall LA Chin N Baribault H Kassner PD Significant gene content variation characterizes the genomes of inbred mouse strains Genome Res 2007 17 12 1743 1745 17989247 10.1101/gr.6754607 PMC2099583

[B5] Graubert TA Cahan P Edwin D Selzer RR Richmond TA Eis PS Shannon WD Li X McLeod HL Cheverud JM Ley TJ A high-resolution map of segmental DNA copy number variation in the mouse genome PLoS Genet 2007 3 1 e3 17206864 10.1371/journal.pgen.0030003 PMC1761046

[B6] Guryev V Saar K Adamovic T Verheul M van Heesch SA Cook S Pravenec M Aitman T Jacob H Shull JD Hubner N Cuppen E Distribution and functional impact of DNA copy number variation in the rat Nat Genet 2008 40 5 538 545 18443591 10.1038/ng.141

[B7] Liu GE Hou Y Zhu B Cardone MF Jiang L Cellamare A Mitra A Alexander LJ Coutinho LL Dell'Aquila ME Gasbarre LC Lacalandra G Li RW Matukumalli LK Nonneman D Regitano LC Smith TP Song J Sonstegard TS Van Tassell CP Ventura M Eichler EE McDaneld TG Keele JW Analysis of copy number variations among diverse cattle breeds Genome Res 2010 20 5 693 703 20212021 10.1101/gr.105403.110 PMC2860171

[B8] Hou Y Liu GE Bickhart DM Cardone MF Wang K Kim ES Matukumalli LK Ventura M Song J VanRaden PM Sonstegard TS Van Tassell CP Genomic characteristics of cattle copy number variations BMC Genomics 2011 12 1 127 21345189 10.1186/1471-2164-12-127 PMC3053260

[B9] Fadista J Nygaard M Holm LE Thomsen B Bendixen C A snapshot of CNVs in the pig genome PLoS One 2008 3 12 e3916 19079605 10.1371/journal.pone.0003916 PMC2596487

[B10] Ramayo-Caldas Y Castelló A Pena RN Alves E Mercadé A Souza CA Fernández AI Perez-Enciso M Folch JM Copy number variation in the porcine genome inferred from a 60 k SNP BeadChip BMC Genomics 2010 11 1 593 20969757 10.1186/1471-2164-11-593 PMC3091738

[B11] Wang X Nahashon S Feaster TK Bohannon-Stewart A Adefope N An initial map of chromosomal segmental copy number variations in the chicken BMC Genomics 2010 11 1 351 20525236 10.1186/1471-2164-11-351 PMC2996973

[B12] Henrichsen CN Chaignat E Reymond A Copy number variants, diseases and gene expression Hum Mol Genet 2009 18 R1 R1 R8 19297395 10.1093/hmg/ddp011

[B13] Zhang F Gu W Hurles ME Lupski JR Copy number variation in human health, disease, and evolution Annu Rev Genomics Hum Genet 2009 10 451 481 19715442 10.1146/annurev.genom.9.081307.164217 PMC4472309

[B14] McCarroll SA Altshuler DM Copy-number variation and association studies of human disease Nat Genet 2007 39 S37 S42 17597780 10.1038/ng2080

[B15] Bronstad I Wolff A Lovas K Knappskog P Husebye E Genome-wide copy number variation (CNV) in patients with autoimmune Addison's disease BMC Med Genet 2011 12 1 111 21851588 10.1186/1471-2350-12-111 PMC3166911

[B16] Ionita-Laza I Rogers AJ Lange C Raby BA Lee C Genetic association analysis of copy-number variation (CNV) in human disease pathogenesis Genomics 2009 93 1 22 26 18822366 10.1016/j.ygeno.2008.08.012 PMC2631358

[B17] Sebat J Lakshmi B Malhotra D Troge J Lese-Martin C Walsh T Yamrom B Yoon S Krasnitz A Kendall J Leotta A Pai D Zhang R Lee YH Hicks J Spence SJ Lee AT Puura K Lehtimäki T Ledbetter D Gregersen PK Bregman J Sutcliffe JS Jobanputra V Chung W Warburton D King MC Skuse D Geschwind DH Gilliam TC Ye K Wigler M Strong association of de novo copy number mutations with autism Science 2007 316 5823 445 449 17363630 10.1126/science.1138659 PMC2993504

[B18] Marklund S Kijas J Rodriguez-Martinez H Rönnstrand L Funa K Moller M Lange D Edfors-Lilja I Andersson L Molecular basis for the dominant white phenotype in the domestic pig Genome Res 1998 8 8 826 833 9724328 10.1101/gr.8.8.826 PMC310759

[B19] Giuffra E Törnsten A Marklund S Bongcam-Rudloff E Chardon P Kijas JMH Anderson SI Archibald AL Andersson L A large duplication associated with dominant white color in pigs originated by homologous recombination between LINE elements flanking KIT Mamm Genome 2002 13 10 569 577 12420135 10.1007/s00335-002-2184-5

[B20] Wright D Boije H Meadows JRS Bed'hom B, Gourichon D, Vieaud A, Tixier-Boichard M, Rubin CJ, Imsland F, Hallböök F, Andersson L: Copy number variation in intron 1 of SOX5 causes the Pea-comb phenotype in chickens PLoS Genet 2009 5 6 e1000512 19521496 10.1371/journal.pgen.1000512 PMC2685452

[B21] Nicholas TJ Cheng Z Ventura M Mealey K Eichler EE Akey JM The genomic architecture of segmental duplications and associated copy number variants in dogs Genome Res 2009 19 3 491 499 19129542 10.1101/gr.084715.108 PMC2661811

[B22] Nicholas TJ Baker C Eichler EE Akey JM A high-resolution integrated map of copy number polymorphisms within and between breeds of the modern domesticated dog BMC Genomics 2011 12 414 21846351 10.1186/1471-2164-12-414 PMC3166287

[B23] Peiffer DA Le JM Steemers FJ Chang W Jenniges T Garcia F Haden K Li J Shaw CA Belmont J Cheung SW Shen RM Barker DL Gunderson KL High-resolution genomic profiling of chromosomal aberrations using Infinium whole-genome genotyping Genome Res 2006 16 9 1136 1148 16899659 10.1101/gr.5402306 PMC1557768

[B24] Winchester L Yau C Ragoussis J Comparing CNV detection methods for SNP arrays Brief Funct Genomic Proteomic 2009 8 5 353 366 19737800 10.1093/bfgp/elp017

[B25] Bae JS Cheong HS Kim LH NamGung S Park TJ Chun JY Kim JY Pasaje CF Lee JS Shin HD Identification of copy number variations and common deletion polymorphisms in cattle BMC Genomics 2010 11 232 20377913 10.1186/1471-2164-11-232 PMC2859865

[B26] Wang K Chen Z Tadesse MG Glessner J Grant SFA Hakonarson H Bucan M Li M Modeling genetic inheritance of copy number variations Nucleic Acids Res 2008 36 21 e138 18832372 10.1093/nar/gkn641 PMC2588508

[B27] Wang K Li M Hadley D Liu R Glessner J Grant SFA Hakonarson H Bucan M PennCNV: an integrated hidden Markov model designed for high-resolution copy number variation detection in whole-genome SNP genotyping data Genome Res 2007 17 11 1665 1674 17921354 10.1101/gr.6861907 PMC2045149

[B28] Smedley D Haider S Ballester B Holland R London D Thorisson G Kasprzyk A BioMart-biological queries made easy BMC Genomics 2009 10 1 22 19144180 10.1186/1471-2164-10-22 PMC2649164

[B29] Ashburner M Ball CA Blake JA Botstein D Butler H Cherry JM Davis AP Dolinski K Dwight SS Eppig JT Harris MA Hill DP Issel-Tarver L Kasarskis A Lewis S Matese JC Richardson JE Ringwald M Rubin GM Sherlock G Gene Ontology: tool for the unification of biology Nat Genet 2000 25 1 25 29 10802651 10.1038/75556 PMC3037419

[B30] Kanehisa M Goto S Furumichi M Tanabe M Hirakawa M KEGG for representation and analysis of molecular networks involving diseases and drugs Nucleic Acids Res 2010 38 suppl 1 D355 D360 19880382 10.1093/nar/gkp896 PMC2808910

[B31] Da Wei Huang BTS Lempicki RA Systematic and integrative analysis of large gene lists using DAVID bioinformatics resources Nat Protoc 2008 4 1 44 57 19131956 10.1038/nprot.2008.211

[B32] Fang M Hu X Jiang T Braunschweig M Hu L Du Z Feng J Zhang Q Wu C Li N The phylogeny of Chinese indigenous pig breeds inferred from microsatellite markers Anim Genet 2005 36 1 7 13 15670125 10.1111/j.1365-2052.2004.01234.x

[B33] Wang JY Guo JF Zhang Q Hu HM Lin HC Wang C Zhang Y Wu Y Genetic Diversity of Chinese Indigenous Pig Breeds in Shandong Province Using Microsatellite Markers Sci 2011 24 1 28 36

[B34] Megens HJ Crooijmans Rp San Cristobal M Hui X Li N Groenen MA Biodiversity of pig breeds from China and Europe estimated from pooled DNA samples: differences in microsatellite variation between two areas of domestication Genet Sel Evol 2008 40 1 103 128 18096118 10.1186/1297-9686-40-1-103 PMC2674914

[B35] Fang M Andersson L Mitochondrial diversity in European and Chinese pigs is consistent with population expansions that occurred prior to domestication Proceedings of the Royal Society B: Biological Sciences 2006 273 1595 1803 1810 10.1098/rspb.2006.3514 PMC1634785 16790414

[B36] Pique-Regi R Monso-Varona J Ortega A Seeger RC Triche TJ Asgharzadeh S Sparse representation and Bayesian detection of genome copy number alterations from microarray data Bioinformatics 2008 24 3 309 318 18203770 10.1093/bioinformatics/btm601 PMC2704547

[B37] Diskin SJ Li M Hou C Yang S Glessner J Hakonarson H Bucan M Maris JM Wang K Adjustment of genomic waves in signal intensities from whole-genome SNP genotyping platforms Nucleic Acids Res 2008 36 19 e126 18784189 10.1093/nar/gkn556 PMC2577347

[B38] Matsuzaki H Wang PH Hu J Rava R Fu GK High resolution discovery and confirmation of copy number variants in 90 Yoruba Nigerians Genome Biol 2009 10 11 R125 19900272 10.1186/gb-2009-10-11-r125 PMC3091319

[B39] Eichler EE Widening the spectrum of human genetic variation Nat Genet 2006 38 1 9 11 16380720 10.1038/ng0106-9

[B40] Conrad DF Andrews TD Carter NP Hurles ME Pritchard JK A high-resolution survey of deletion polymorphism in the human genome Nat Genet 2006 38 1 75 81 16327808 10.1038/ng1697

[B41] Freeman JL Perry GH Feuk L Redon R McCarroll SA Altshuler DM Aburatani H Jones KW Tyler-Smith C Hurles ME Carter NP Scherer SW Lee C Copy number variation: new insights in genome diversity Genome Res 2006 16 8 949 961 16809666 10.1101/gr.3677206

[B42] Conrad DF Hurles ME The population genetics of structural variation Nat Genet 2007 39 S30 S36 17597779 10.1038/ng2042 PMC2716079

[B43] Hasin Y Olender T Khen M Gonzaga-Jauregui C Kim PM Urban AE Snyder M Gerstein MB Lancet D Korbel JO High-resolution copy-number variation map reflects human olfactory receptor diversity and evolution PLoS Genet 2008 4 11 e1000249 18989455 10.1371/journal.pgen.1000249 PMC2570968

[B44] Young JM Endicott RLM Parghi SS Walker M Kidd JM Trask BJ Extensive copy-number variation of the human olfactory receptor gene family Am J Hum Genet 2008 83 2 228 242 18674749 10.1016/j.ajhg.2008.07.005 PMC2495065

[B45] Ramos AM Crooijmans RP Affara NA Amaral AJ Archibald AL Beever JE Bendixen C Churcher C Clark R Dehais P Hansen MS Hedegaard J Hu ZL Kerstens HH Law AS Megens HJ Milan D Nonneman DJ Rohrer GA Rothschild MF Smith TP Schnabel RD Van Tassell CP Taylor JF Wiedmann RT Schook LB Groenen MA Design of a high density SNP genotyping assay in the pig using SNPs identified and characterized by next generation sequencing technology PLoS One 2009 4 8 e6524 19654876 10.1371/journal.pone.0006524 PMC2716536

[B46] Bickhart DM Hou Y Schroeder SG Alkan C Cardone MF Matukumalli LK Song J Schnabel RD Ventura M Taylor JF Garcia JF Van Tassell CP Sonstegard TS Eichler EE Liu GE Copy number variation of individual cattle genomes using next-generation sequencing Genome res 2012 22 4 778 790 22300768 10.1101/gr.133967.111 PMC3317159

[B47] Castle JC Biery M Bouzek H Xie T Chen R Misura K Jackson S Armour CD Johnson JM Rohl CA Raymond CK DNA copy number, including telomeres and mitochondria, assayed using next-generation sequencing BMC Genomics 2010 11 1 244 20398377 10.1186/1471-2164-11-244 PMC2867831

[B48] Liu GE Brown T Hebert DA Cardone MF Hou Y Choudhary RK Shaffer J Amazu C Connor EE Ventura M Gasbarre LC Initial analysis of copy number variations in cattle selected for resistance or susceptibility to intestinal nematodes Mamm Genome 2011 22 111 121 21125402 10.1007/s00335-010-9308-0

[B49] Livak KJ Schmittgen TD Analysis of relative gene expression data using real-time quantitative PCR and the 2-[Delta][Delta] CT method Methods 2001 25 4 402 408 11846609 10.1006/meth.2001.1262

[B50] Ballester M Castelló A Ibáez E Sánchez A Folch JM Real-time quantitative PCR-based system for determining transgene copy number in transgenic animals Biotechniques 2004 37 4 610 613 15517974 10.2144/04374ST06

[B51] Karolchik D Kuhn RM Baertsch R Barber GP Clawson H Diekhans M Giardine B Harte RA Hinrichs AS Hsu F Kober KM Miller W Pedersen JS Pohl A Raney BJ Rhead B Rosenbloom KR Smith KE Stanke M Thakkapallayil A Trumbower H Wang T Zweig AS Haussler D Kent WJ The UCSC genome browser database: 2008 update Nucleic Acids Res 2008 36 suppl 1 D773 D779 18086701 10.1093/nar/gkm966 PMC2238835

[B52] Kurreeman FA Goulielmos GN Alizadeh BZ Rueda B Houwing-Duistermaat J Sanchez E Bevova M Radstake TR Vonk MC Galanakis E Ortego N Verduyn W Zervou MI Roep BO Dema B Espino L Urcelay E Boumpas DT van den Berg LH Wijmenga C Koeleman BP Huizinga TW Toes RE Martin J AADEA Group SLEGEN Consortium The TRAF1-C5 region on chromosome 9q33 is associated with multiple autoimmune diseases Ann Rheum Dis 2010 69 4 696 699 19433411 10.1136/ard.2008.106567

[B53] Jawaheer D Seldin MF Amos CI Chen WV Shigeta R Monteiro J Kern M Criswell LA Albani S Nelson JL Clegg DO Pope R Schroeder HW Bridges SL Pisetsky DS Ward R Kastner DL Wilder RL Pincus T Callahan LF Flemming D Wener MH Gregersen PK A genomewide screen in multiplex rheumatoid arthritis families suggests genetic overlap with other autoimmune diseases Am J Hum Genet 2001 68 4 927 936 11254450 10.1086/319518 PMC1275647

[B54] Redler S Brockschmidt FF Forstbauer L Giehl KA Herold C Eigelshoven S Hanneken S De Weert J Lutz G Wolff H Kruse R Blaumeiser B Böhm M Becker T Nöthen MM Betz RC The TRAF1/C5 locus confers risk for familial and severe alopecia areata Br J Dermatol 2010 162 4 866 869 20030635 10.1111/j.1365-2133.2009.09598.x

[B55] Renard M Holm T Veith R Callewaert BL Adès LC Baspinar O Pickart A Dasouki M Hoyer J Rauch A Trapane P Earing MG Coucke PJ Sakai LY Dietz HC De Paepe AM Loeys BL Altered TGFβ signaling and cardiovascular manifestations in patients with autosomal recessive cutis laxa type I caused by fibulin-4 deficiency Eur J Hum Genet 2010 18 8 895 901 20389311 10.1038/ejhg.2010.45 PMC2987390

[B56] Zhang W Xu C Bian C Tempel W Crombet L MacKenzie F Min J Liu Z Qi C Crystal structure of the Cys2His2-type zinc finger domain of human DPF2 Biochem Biophys Res Commun 2010 413 1 58 61 21888896 10.1016/j.bbrc.2011.08.043

[B57] Davis CB Littman DR Thymocyte lineage commitment: is it instructed to stochastic? Curr Opin Immunol 1994 6 2 266 272 7912080 10.1016/0952-7915(94)90100-7

[B58] Killeen N Davis CB Chu K Crooks MEC Sawada S Scarborough JD Boyd KA Stuart SG Xu H Littman DR CD4 function in thymocyte differentiation and T cell activation. Philosophical Transactions Biological Sciences 1993 25 34 7904343 10.1098/rstb.1993.0131

[B59] Butterfield DA Hardas SS Lange MLB Oxidatively modified glyceraldehyde-3-phosphate dehydrogenase (GAPDH) and Alzheimer's disease: many pathways to neurodegeneration J Alzheimers Dis 2010 20 2 369 393 20164570 10.3233/JAD-2010-1375 PMC2922983

[B60] Chuang DM Hough C Senatorov VV Glyceraldehyde-3-phosphate dehydrogenase, apoptosis, and neurodegenerative diseases Annu Rev Pharmacol Toxicol 2005 45 269 290 15822178 10.1146/annurev.pharmtox.45.120403.095902

[B61] Vidal R Ghetti B Takao M Brefel-Courbon C Uro-Coste E Glazier BS Siani V Benson MD Calvas P Miravalle L Rascol O Delisle MB Intracellular ferritin accumulation in neural and extraneural tissue characterizes a neurodegenerative disease associated with a mutation in the ferritin light polypeptide gene J Neuropathol Exp Neurol 2004 63 4 363 380 15099026 10.1093/jnen/63.4.363

[B62] Girelli D Corrocher R Bisceglia L Olivieri O De Franceschi L Zelante L Gasparini P Molecular basis for the recently described hereditary hyperferritinemia-cataract syndrome: a mutation in the iron-responsive element of ferritin L-subunit gene (the" Verona mutation") Blood 1995 86 11 4050 4053 7492760

[B63] Sato M Taniguchi T Tanaka N The interferon system and interferon regulatory factor transcription factors-studies from gene knockout mice Cytokine Growth Factor Rev 2001 12 2–3 133 142 11325597 10.1016/s1359-6101(00)00032-0

[B64] Taniguchi T Takaoka A The interferon-[alpha]/[beta] system in antiviral responses: a multimodal machinery of gene regulation by the IRF family of transcription factors Curr Opin Immunol 2002 14 1 111 116 11790540 10.1016/s0952-7915(01)00305-3

[B65] Genome-wide association study of 14,000 cases of seven common diseases and 3,000 shared controls Nature 2007 447 7145 661 678 17554300 10.1038/nature05911 PMC2719288

